# Reference Values of Skeletal Muscle Mass for Korean Children and Adolescents Using Data from the Korean National Health and Nutrition Examination Survey 2009-2011

**DOI:** 10.1371/journal.pone.0153383

**Published:** 2016-04-13

**Authors:** Kirang Kim, Sangmo Hong, Eun Young Kim

**Affiliations:** 1 Department of Food Science and Nutrition, Dankook University, Yongin, Republic of Korea; 2 Department of Endocrinology and Metabolism, Hanyang University Guri Hospital, Gyeonggi-do, Republic of Korea; 3 Department of Radiology, Gachon University Gil Hospital, Incheon, Republic of Korea; Baylor College of Medicine, UNITED STATES

## Abstract

**Background:**

Skeletal muscle mass (SMM) plays a crucial role in systemic glucose metabolism.

**Objective:**

To obtain reference data on absolute and relative values of SMM for Korean children and adolescents.

**Methods:**

Cross-sectional results from 1919 children and adolescents (1024 boys) aged 10–18 years that underwent dual-energy X-ray absorptiometry (DXA) during the Korean National Health and Nutrition Examination Survey 2009–2011 were analyzed. SMMs were evaluated as follows; absolute SMM (appendicular skeletal muscle mass [ASM]) and relative SMMs, namely, height-adjusted skeletal muscle index (SMI; ASM/height^2^), %SMM (ASM/weight x 100), and skeletal muscle-to-body fat ratio (MFR; ASM/body fat mass).

**Results:**

Percentile curves illustrated the developmental patterns of the SMMs of Korean children and adolescents. ASM and SMI increased with age in both genders, and increased from age 10 throughout adolescence in boys, whereas in girls, they increased until age 13 and then stabilized. In boys, %SMM and MFR were highest at age 15 and then slowly stabilized or decreased, whereas in girls, they peaked at age 10 to 11 and then decreased through adolescence. Cut-off values for low MFR were identified and a significant association was found between a low MFR and high risk of metabolic syndrome. However, this association was found to be dependent on gender and the level of BMI.

**Conclusion:**

This study provides reference values of absolute and relative SMM for Korean children and adolescents. Detailed body composition analyses including skeletal muscle and fat mass might provide improved measures of metabolic risk.

## Introduction

Skeletal muscle mass (SMM) importantly affects locomotion and the maintenance of posture, and as the most abundant insulin-sensitive tissue, plays a crucial role in systemic glucose metabolism. Decreased muscle mass, known as sarcopenia, typically accompanies the aging process and is a major cause of physical disability, loss of independence, and frailty [1]. However, the problem of low muscle mass extends beyond the elderly; sarcopenia is a risk factor of insulin resistance and is associated with metabolic risk in children and adolescents [2, 3]. Furthermore, elevated body fat levels may act in synergism with decreased SMM, because adiposity is also closely linked to insulin resistance [4], and thus, ratios of SMM and measures of fatness, such as, skeletal muscle-to-body fat ratio (MFR) could importantly predict the development of metabolic disease [5]. However, the use of SMM as a surveillance tool is limited by the absence of reference data regarding SMM across the age spectrum for children and adolescents. With rising recognition for the importance of SMM, a recent study [6] revealed reference percentile curves of bioelectrical impedance analysis (BIA) determined SMM and suggested cut-off value of MFR to determine sarcopenia for British children and adolescents.

A wide range of techniques, such as, magnetic resonance imaging (MRI), computed tomography (CT), dual energy X-ray absorptiometry (DXA), and BIA, and ratios of total or partial body potassium to fat-free soft tissue mass can be used to assess SMM. CT and MRI are regarded as reference standards for the analysis of body composition, because the precise imaging studies can be used to differentiate fat and SMM from other soft tissues [7, 8]. However, DXA and BIA are more accessible in clinical practice for availability and cost reasons; whole-body DXA provides accurate measures of whole-body bone mineral content (BMC), fat-free mass, and fat mass, which allow absolute and relative values of SMM to be calculated [9].

To the best of our knowledge, no previous study has specifically sought to determine SMM reference values for Korean children and adolescents. Accordingly, the purpose of this study was to obtain absolute and relative SMM reference data and to suggest MFR cut-off values for the determination of sarcopenia in Korean children and adolescents.

## Methods

The study protocol was reviewed and approved by the institutional review board of the Korean Center for Disease Control and Prevention (KCDC)’s (2009-01CON-03-2C, 2010-02CON- 21-C), and written consent was obtained from all subjects. Further ethical approval for the use of open Korea National Health and Nutrition Examination Survey (KNHANES) data was exempted from institutional review board approval since publicly available data was used in the present study.

### Study population

The KNHANES data of Korean children and adolescents aged from 10 to 18 years that underwent a DXA examination between January 2009 and May 2011 was analyzed. KNHANES is a nationwide cross-sectional survey that has been performed in Korea since 1998 by the KCDC. The survey used a stratified multi-stage clustered probability sampling procedure to select a representative sample of non-institutionalized members of the civilian Korean population [10].

### Anthropometric and laboratory measurements

Anthropometric measurements (height, weight, and waist circumference) obtained during the health examination were used. Body mass index (BMI) was defined as weight divided by height squared (kg/m^2^). Height was measured using a stadiometer (SECA, Hamburg, Germany) and weight using a balance beam scale (G-TECH, Uijeongbu, Korea) with participants wearing a standardized gown. Blood pressure (BP) was measured with subjects in a sitting position after a 5-minute rest period. Systolic and diastolic BPs were measured by placing a mercury sphygmomanometer (Baumanometer, W.A. Baum Co., Copiague, NY, USA) placed on right arms. Blood samples were taken by a skilled nurse in a mobile vehicle and transported daily to a Central Laboratory (NEODIN Medical Institute, Seoul, Korea). Serum triglyceride and HDL cholesterol were determined enzymatically using a chemistry analyzer (Hitachi 7600, Japan), and a glucose oxidase method was used to measure plasma glucose.

### Dual-energy X-ray absorptiometry measurements

For KNHANES, whole body DXA examinations were conducted using a QDR Discovery (formerly, the QDR 4500A) fan beam densitometer (Hologic, Inc., Bedford, MA, USA), according to the manufacturer’s instructions. The DXA scanner was calibrated daily using a spine phantom and weekly using a step phantom. DXA examinations were performed with subjects wearing light clothing; any item that could have possibly interfered with results was removed.

A whole body DXA scan was performed on each subject to measure BMC (g), bone mineral density (BMD, g/cm2), fat mass (g), and fat-free mass including BMC (g), along with demographic information. Absolute SMM (appendicular skeletal muscle mass [ASM]), which is regarded a good proxy for total body SMM [11], was calculated as the sum of muscle mass in arms and legs, assuming that all non-fat and non-bone tissue is skeletal muscle. The following relative SMM values were defined as follows; skeletal muscle index (SMI) adjusted by height = ASM (kg)/height (m) ^2^, %SMM = ASM (kg)/weight (kg) x 100, and MFR = ASM (kg)/body fat mass (kg) [5].

### Metabolic syndrome components

Metabolic syndrome (MS) risk components for children ages 10 or older were defined using the criteria established by the International Diabetes Federation (IDF) [12]; (i) high BP = systolic BP ≥ 130 mmHg or diastolic BP ≥ 85 mmHg, (ii) elevated fasting blood glucose (≥ 5.6 mmol/L) (iii) hypertriglyceridemia (≥ 1.7 mmol/L), (iv) low serum HDL cholesterol (< 1.03 mmol/L), and (v) abdominal obesity was defined as the waist circumference ≥ 90^th^ percentile for age and sex for 10–16 yr, and waist circumference ≥ 90 cm for male adolescents and ≥ 80 cm for female adolescents of 17 yr and 18 yr [13].

### Statistical analysis

Descriptive statistics were calculated for anthropometric measures and results are expressed as means ± standard deviations (SDs). Smoothed percentile curves for SMMs were constructed for boys and girls separately using the Lambda-Mu-Sigma (LMS) method (LMSChartMaker Pro Version 2.54, Medical Research Council, London, UK) [14], which summarizes the changing distribution by three curves representing the skewness expressed as a Box–Cox power (L), the median (M) and coefficient of variation (S). LMS model fitting was performed using sample weights obtained from KNHANES data sets and the method assumed that the data can be normalized by using power transformation. Final percentile curves were chosen by selecting more parsimonious models over more complex models based on deviance measurements of models satisfying goodness of fit based on Q tests. Q statistic was considered adequate if Q statistics curves for L, M, and S were within the range -2 to +2 [15].

Sarcopenia was originally described in elderly individuals, and because the cut-off points used to define sarcopenia are based on the DXA-determined SMMs of healthy young reference groups (ages between 20–40 years) in epidemiologic studies, the cut-offs used to define sarcopenia inevitably have ethnic and gender specific characteristics [16]. Furthermore, the diagnostic criteria for sarcopenia have not been established in children and adolescents, because they have growing body which make difficult to set uniform cut-off values for all ages combined for this population. Accordingly, the cut-off value of MFR was evaluated to determine sarcopenia on present study. The MFR values expressed as histograms for each gender separately and values were compared with respect to BMI level. The BMI level was categorized into two groups; a normal group (BMI < 85^th^ percentile for age and sex) and an at risk of overweight and obesity group (BMI ≥ 85^th^ percentile for age and sex) based on the Standard Growth Charts of Korean children and adolescents in 2007 published by the KCDC and Korean Pediatric Society [13]. In addition, according to the previous methodology to define sarcopenia [6], each gender was divided into quintiles of BMI z-score and the mean and SD of MFR were calculated for each quintile. A cut-off values were defined using mean and SD of MFR for the 3^rd^ BMI quintile (ie, cut-off value = mean value– 2SD of MFR for the 3^rd^ BMI quintile), and the proportions of sarcopenic subjects were examined.

The odds ratio (OR) for one or more MS risk components were analyzed according to the BMI level and presence of sarcopenia based on the determined MFR cut-off values. The p values of < 0.05 were considered statistically significant. Data processing and analysis were performed using a commercially available software program (PASW, version 17.0; SPSS, Chicago, IL, USA).

## Results

### Body composition characteristics by gender and age

A total of 1919 children and adolescents (1024 boys) were included in the study. Table 1 summarizes mean values of height, weight, BMI, fat mass, fat-free mass, fat mass index (FMI, fat mass/height^2^), fat-free mass index (FFMI; fat-free mass/height^2^), ASM, and relative values of SMM by gender and age. Generally, height, weight and fat-free mass were higher for boys but fat mass was higher for girls from 11 years of age (all p values < 0.01); fat mass was similar in 10-year-old boys and girls.

**Table 1:** Anthropometric and body composition characteristics by sex and age.

Age (yr)	No.	Height (cm)	Weight (kg)	BMI (kg/m^2^)	Fat mass (kg)	Fat-free mass (kg)	FMI (kg/m^2^)	FFMI (kg/m^2^)	ASM (kg)	SMI (ASM/Ht^2^)	%SMM (ASM/Wt X 100)	**MFR (ASM/fat mass)**
**Boys**												
**10**	118	142.3 ± 0.7	38.8 ± 1.1	19.0 ± 0.4	11.4 ± 0.7	25.8 ± 0.5	5.6 ± 0.3	12.7± 0.1	11.2 ± 0.2	5.5 ± 0.1	29.5 ± 0.4	1.2 ± 0.1
**11**	127	149.0 ± 0.7	45.0 ± 1.1	20.1 ± 0.4	13.5 ± 0.7	29.8 ± 0.5	6.0 ± 0.3	13.3 ± 0.2	13.2 ± 0.3	5.9± 0.1	29.8 ± 0.4	1.2 ± 0.1
**12**	133	156.3 ± 0.7	49.4 ± 1.1	20.1 ± 0.4	13.6 ± 0.8	33.8 ± 0.6	5.5 ± 0.3	13.7 ± 0.1	15.4 ± 0.3	6.2 ± 0.1	31.6 ± 0.5	1.4 ± 0.1
**13**	109	162.2 ± 1.0	54.7± 1.3	20.7 ± 0.4	13.4 ± 0.7	39.0 ± 0.7	5.1 ± 0.3	14.7 ± 0.2	17.9 ± 0.4	6.8 ± 0.1	33.2 ± 0.5	1.6 ± 0.1
**14**	143	169.4 ± 0.6	61.6 ± 1.3	21.4 ± 0.4	14.2 ± 0.8	44.6 ± 0.7	4.9 ± 0.3	15.5 ± 0.2	20.5 ± 0.3	7.1 ± 0.1	33.9 ± 0.4	1.9 ± 0.1
**15**	106	171.7 ± 0.8	61.6 ± 1.4	20.7 ± 0.4	12.5 ± 0.8	46.1 ± 0.8	4.2 ± 0.2	15.6 ± 0.2	21.4 ± 0.4	7.2 ± 0.1	35.2 ± 0.4	2.1 ± 0.1
**16**	94	173.8 ± 0.6	65.5 ± 1.6	21.6 ± 0.5	13.9 ± 0.9	48.5 ± 0.8	4.6 ± 0.3	16.0 ± 0.2	22.2 ± 0.4	7.3 ± 0.1	34.3 ± 0.4	2.0 ± 0.1
**17**	107	173.8 ± 0.7	65.9 ± 1.2	21.7 ± 0.4	13.8 ± 0.7	48.9 ± 0.8	4.6 ± 0.2	16.2 ± 0.2	22.4 ± 0.4	7.4 ± 0.1	34.2 ± 0.3	1.9 ± 0.1
**18**	87	173.2 ± 0.9	65.2 ± 1.2	21.8 ± 0.4	13.6 ± 0.7	48.5 ± 0.7	4.5 ± 0.2	16.2 ± 0.2	22.0 ± 0.3	7.3 ± 0.1	33.9 ± 0.3	1.9 ± 0.1
**Girls**												
**10**	108	143.8 ± 0.9	37.1 ± 0.9	17.8 ± 0.3	11.5 ± 0.5	24.2 ± 0.5	5.5 ± 0.2	11.6 ± 0.1	10.3 ± 0.2	5.0 ± 0.1	28.0 ± 0.3	1.0 ± 0.0
**11**	111	150.3 ± 0.7	42.5 ± 1.0	18.7 ± 0.3	13.0 ± 0.5	27.8 ± 0.5	5.7 ± 0.2	12.2 ± 0.2	12.0 ± 0.3	5.3 ± 0.1	28.5 ± 0.3	1.0 ± 0.0
**12**	95	155.8 ± 0.7	47.6 ± 1.2	19.5 ± 0.4	14.2 ± 0.6	31.4 ± 0.6	5.8 ± 0.2	12.9 ± 0.2	13.5 ± 0.3	5.5 ± 0.1	28.7 ± 0.3	1.1 ± 0.0
**13**	123	157.8 ± 0.7	50.6 ± 0.9	20.2 ± 0.3	15.7 ± 0.6	32.6 ± 0.5	6.3 ± 0.2	13.1 ± 0.1	13.9 ± 0.3	5.5 ± 0.1	27.6 ± 0.3	1.0 ± 0.0
**14**	106	159.6 ± 0.5	53.0 ± 1.0	20.8 ± 0.4	17.6 ± 0.6	33.0 ± 0.4	6.9 ± 0.2	12.9 ± 0.2	14.0 ± 0.2	5.5 ± 0.1	26.5 ± 0.3	0.8 ± 0.0
**15**	89	160.7 ± 0.6	53.4 ± 1.3	20.6 ± 0.5	17.6 ± 0.9	33.5 ± 0.6	6.8 ± 0.3	12.9 ± 0.2	14.0 ± 0.3	5.4 ± 0.1	26.4 ± 0.3	0.9 ± 0.0
**16**	97	159.8 ± 0.6	54.7 ± 1.2	21.4 ± 0.4	18.3 ± 0.7	34.1 ± 0.5	7.1 ± 0.3	13.3 ± 0.2	14.5 ± 0.3	5.7 ± 0.1	26.6 ± 0.3	0.8 ± 0.0
**17**	99	161.6 ± 0.6	56.7 ± 1.7	21.7 ± 0.6	19.4 ± 1.1	34.9 ± 0.7	7.4 ± 0.4	13.3 ± 0.2	14.6 ± 0.3	5.6 ± 0.1	26.0 ± 0.3	0.8 ± 0.0
**18**	67	161.1 ± 0.9	53.9 ± 1.3	20.7 ± 0.4	17.6 ± 0.8	34.0 ± 0.6	6.8 ± 0.3	13.1 ± 0.2	14.1 ± 0.3	5.4 ± 0.1	26.3 ± 0.3	0.9 ± 0.0

Note- Data are means ± standard deviations, BMI, body mass index; FMI, fat mass index; FFMI, fat-free mass index; ASM, appendicular skeletal muscle mass; SMI, skeletal muscle index; SMM, skeletal muscle mass; Ht, height; Wt, weight; MFR, skeletal muscle to body fat ratio.

**Fig 1:**
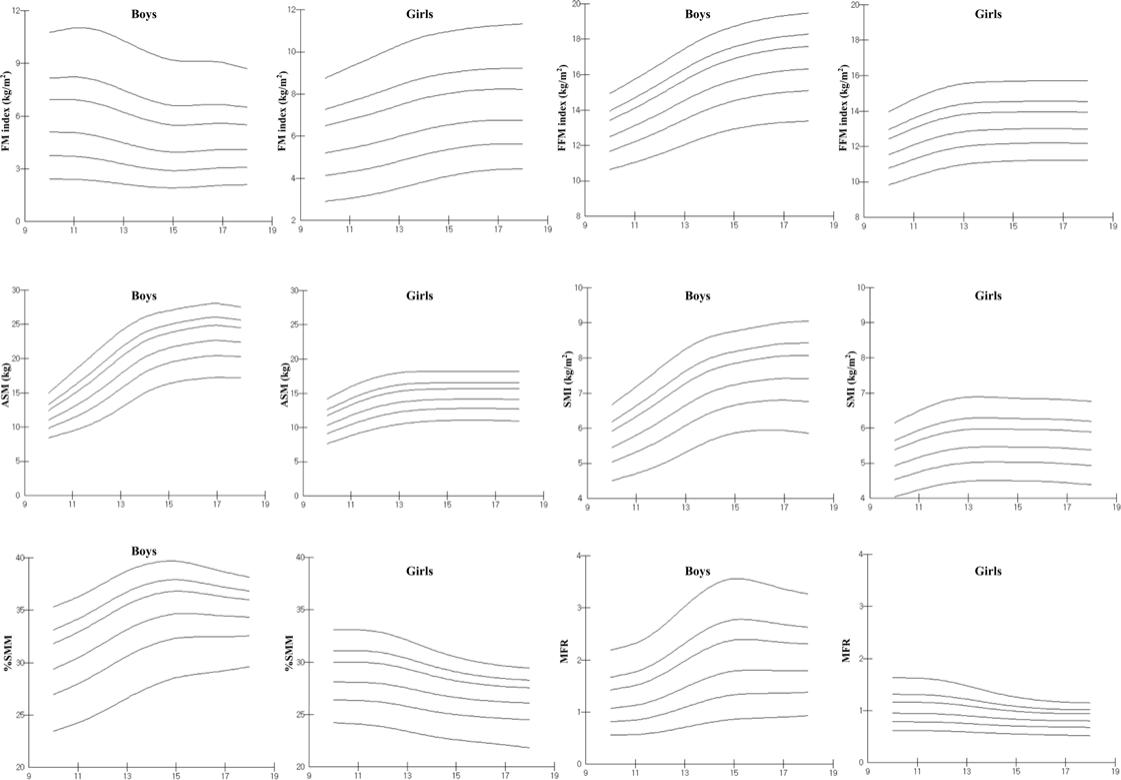
Percentile curves of skeletal muscle mass. Curves of the 5^th^, 25^th^, 50^th^, 75^th^, 85^th^, and 95^th^ percentiles for fat mass index (FM index), fat-free mass index (FFM index), appendicular skeletal muscle mass (ASM), skeletal muscle index (SMI), %SMM, and skeletal muscle to body fat ratio (MFR) by sex and age.

Smoothed LMS curves for the 5^th^, 25^th^, 50^th^, 75^th^, 85^th^, and 95^th^ percentiles of FMI, FFMI, ASM, and relative values of SMM for boys and girls are presented in Fig 1. For boys, FMI peaked at age 11, decreased until age 15, and then stabilized, whereas FFMI was lowest at age 10 and gradually increased through adolescence. For girls, FMI increased rapidly from age 10 to 14, and FFMI increased from aged 10 to age 13 and then stabilized.

Skeletal muscle mass indices demonstrated different age-related growth patterns for genders; ASM increased with age from 10 to 18 years old for boys and girls; the 50^th^ percentile increased from 11.0 kg to 22.4 kg over this period for boys and from 10.31 kg to 14.1 kg for girls. In boys, ASM variability increased across the age spectrum, whereas in girls, ASM variability peaked at around age 13 and then decreased. Percentile curves for SMI were similar to FFMI curves; in boys, SMI 50^th^ percentiles increased from 5.45 at age 10 to 7.24 at age 15 and then remained relatively unchanged, whereas in girls, percentiles increased from 4.92 at age 10 to 5.45 at age 13, and then remained stable or decreased slightly until age 18. The percentile curve patterns for %SMM were similar to those of MFR, and also revealed different gender growth patterns; for boys, %SMM and MFR were highest at age 15 (50^th^ percentiles for %SMM and MFR were 34.66 and 1.79, respectively) and then slowly decreased or remained flat, whereas for girls, they were highest at age 10 and then decreased through adolescence, particularly between 12 and 14 years.

### Relations between MFR and BMI

**Fig 2:**
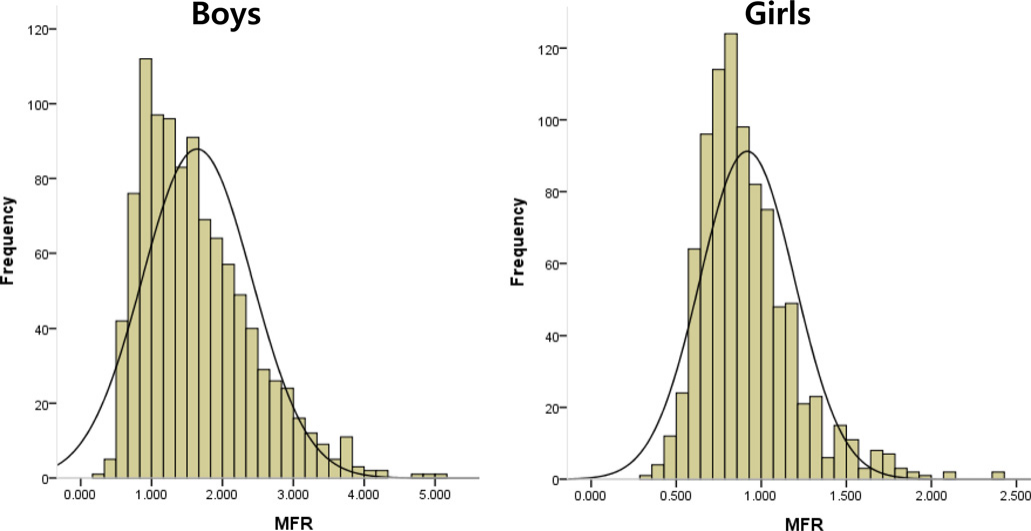
Histogram of skeletal muscle to body fat ratio (MFR) for all 1919 study subjects.

The MFR distribution of all 1919 study subjects (boys and girls of all ages) is provided as a histogram in Fig 2. For the 1024 boys, mean (SD) MFR was 1.710 (0.808) and ranged between 0.313 and 5.042, and for the 885 girls, mean (SD) MFR was 0.908 (0.269) and ranged between 0.291 and 2.410.

Table 2 provides MFR values for different BMI levels. Mean MFR was significantly higher for normal subjects than overweight or obese subjects (all p values < 0.01), and the MFR difference between normal and overweight or obese subjects was larger for boys than girls.

**Table 2:** Skeletal muscle to body fat ratio (MFR) by sex, age and body mass index (BMI).

Age	MFR for normal BMI (BMI < 85^th^ percentile for age and sex)	MFR for overweight and obesity (BMI ≥ 85^th^ percentile for age and sex)	*P* value
**Boys**			
**10 yr**	1.36 ± 0.44	0.68 ± 0.13	< 0.001
**11 yr**	1.30 ± 0.51	0.71 ± 0.16	< 0.001
**12 yr**	1.57 ± 0.67	0.77 ± 0.18	< 0.001
**13 yr**	1.80 ± 0.68	0.92 ± 0.18	< 0.001
**14 yr**	2.08 ± 0.92	1.05 ± 0.38	< 0.001
**15 yr**	2.27 ± 0.77	0.99 ± 0.18	< 0.001
**16 yr**	2.21 ± 0.77	1.03 ± 0.21	< 0.001
**17 yr**	2.02 ± 0.73	1.19 ± 0.34	< 0.001
**18 yr**	1.99 ± 0.69	1.19 ± 0.28	< 0.001
**total**	1.88 ± 0.78	0.94 ± 0.30	< 0.001
**Girls**			
**10 yr**	1.02 ± 0.27	0.70 ± 0.10	< 0.001
**11 yr**	1.09 ± 0.31	0.71 ± 0.12	< 0.001
**12 yr**	1.13 ± 0.34	0.79 ± 0.14	< 0.001
**13 yr**	1.04 ± 0.30	0.69 ± 0.14	< 0.001
**14 yr**	0.90 ± 0.20	0.68 ± 0.16	< 0.001
**15 yr**	0.89 ± 0.19	0.66 ± 0.14	< 0.001
**16 yr**	0.88 ± 0.17	0.65 ± 0.13	< 0.001
**17 yr**	0.85 ± 0.17	0.64 ± 0.17	< 0.001
**18 yr**	0.92 ± 0.20	0.54 ± 0.12	< 0.001
**total**	0.96 ± 0.26	0.68 ± 0.15	< 0.001

Note- Data are means ± standard deviations.

As shown in Table 3, the cut-off value for sarcopenia (mean MFR– 2SD of the 3^rd^ BMI quintile) was 0.374 for boys and 0.554 for girls and the proportions below these cut-offs were 0.1% for boys and 3.8% for girls (5.0% for boys and 16.2% for girls of the highest BMI quintile). When we reset the cut-off value for sarcopenia as mean MFR– 1SD of the 3^rd^ BMI quintile, the cut-off values were 1.155 and 0.723 and the proportions of sarcopenia were 32.1% and 24.3% for boys and girls (71.2% for boys and 59.8% for girls of the highest BMI quintile), respectively.

**Table 3:** Skeletal muscle to body fat ratio (MFR) across body mass index (BMI) z-score quintiles.

Boys					Girls				
BMI	zBMI	MFR	class I* (%)	class II** (%)	BMI	zBMI	MFR	class I^†^ (%)	class II^††^ (%)
**Q1**	-1.273(0.238)	2.081 (0.727)	17 (8.3)^§^	0^§^	**Q1**	-1.224 (0.293)	1.182 (0.312)	7 (3.9)^§^	0^§^
**Q2**	-0.649 (0.145)	2.103 (0.806)	35 (17.1)	0	**Q2**	-0.608 (0.120)	1.001 (0.240)	15 (8.4)	0
**Q3**	-0.179 (0.140)	1.936 (0.781)	46 (22.4)	0	**Q3**	-0.171 (0.124)	0.892 (0.169)	26 (14.5)	0
**Q4**	0.421 (0.212)	1.462 (0.722)	85 (41.5)	0	**Q4**	0.340 (0.188)	0.815 (0.153)	60 (33.5)	5 (2.8)
**Q5**	1.494 (0.660)	1.040 (0.342)	146 (71.2)	1 (5)	**Q5**	1.511 (0.764)	0.681 (0.144)	107 (59.8)	29 (16.2)
**Total**		1.710 (0.808)	329 (32.1)	1(0.1)	**Total**		0.908 (0.269)	215(24.3)	34 (3.8)

Results are presented as means (standard deviations).

* The class I MFR cut-off for boys (mean - 1SD of MFR for the 3^rd^ BMI quintile) to determine sarcopenia = 1.155.

** The class II MFR cut-off for boys (mean - 2SDs of MFR for the 3^rd^ BMI quintile) to determine sarcopenia = 0.374.

^†^ The class I MFR cut-off for girls (mean - 1SD of MFR for the 3^rd^ BMI quintile) to determine sarcopenia = 0.723.

^††^ The class II MFR cut-off for girls (mean - 2SDs of MFR for the 3^rd^ BMI quintile) to determine sarcopenia = 0.554.

^§^ The proportion below each cut-off.

### Odds ratios of MS risk components as determined using MFR and BMI levels

A total of 572 subjects (304 boys) had one or more of MS risk components. Table 4 shows ORs for the presence of MS components according the presence of sarcopenia. Risks of MS were significantly higher in subjects with sarcopenia (OR, 2.67; 95% CI, 2.05–3.49, p < 0.01); the increased ORs were observed in both genders. When study subjects with a normal BMI and MFR were used as the reference group, ORs of MS risk were found to be significantly increased in subjects with a high BMI and a normal MFR (OR, 4.59; 95% CI, 2.94 to 7.17, p < 0.01), and in those with a high BMI and sarcopenia (OR, 8.28; 95% CI, 5.60 to 11.45, p < 0.01), and ORs were greater for girls than boys. On the other hand, for boys and girls in the normal BMI group, the association between MS risk and sarcopenia was nonsignificant (p = 0.07).

**Table 4:** Odds ratios of presence of metabolic risk components by skeletal muscle to body fat ratio (MFR) and body mass index (BMI).

	All BMI		normal BMI(BMI<85^th^)		high BMI (BMI≥85^th^)	
	Normal MFR	Sarcopenia	Normal MFR	Sarcopenia	Normal MFR	Sarcopenia
**Total**	1.00	2.67	1.00	1.12	4.59	8.28
		(2.05–3.49)		(0.76–1.66)	(2.94–7.17)	(5.60–11.45)
**Boys**	1.00	2.33	1.00	0.87	4.69	6.95
		(1.70–3.19)		(0.52–1.46)	(2.17–10.12)	(4.73–10.22)
**Girls**	1.00	3.20	1.00	1.55	4.81	10.48
		(2.10–4.90)		(0.89–2.68)	(2.79–8.27)	(6.04–18.20)

Note- using the MFR cut-off as 1.155 for boys and 0.723 for girls.

## Discussion

This study provides reference growth data for SMM indices for Korean children and adolescents. In particular, it provides age and gender-specific percentile curves for SMM, including absolute (ASM) and relative values of SMM (SMI, %SMM, MFR) based on a nationally representative KNHANES data set acquired using DXA technology. Recently, McCarthy et al. reported reference values for SMM (ASM, %SMM, and MFR) for Caucasian children aged 5–18 years (n = 1985) in the UK using BIA [6]. The growth patterns of SMM in children and adolescents were found to be similar in this UK study and in the present study, that is, ASM increased with age in boy and girls and age-related variability was greater for boys. However, the percentile curve patterns of % SMM and MFR differed between genders of two ethnics; % SMM and MFR gradually increase until age 15 and then slowly decreased or remained flat for Korean boys, whereas they peaked at age 10–11 and then decrease through adolescence in Korea girls. Interestingly, in the UK, % SMM and MFR gradually increased through adolescence from age 10 in both girls and boys [6]. In addition, older Korean adolescents (17 and 18 year olds) were found to be less muscular than UK adolescents (mean ASM 18.9 kg for Korean boys vs. 24.8 kg for UK boys, and 13.5 kg for Korean girls vs. 17.5 kg for UK girls). Furthermore, MFR variability was greater for Korean boys than UK boys (mean ± SD and range, 1.71 ± 0.81 and 0.31–5.04 for Korean boys at aged 10–18, and 1.99 ± 0.60, 0.69–4.84 for UK boys aged 5–18 year), which in the previous method [6], lead to determine sarcopenia inadequate to adopt in our population. To identify MFR cut-off to determine sarcopenia; they introduced the cut-off values for low MFR as the mean value—2 SD of MFR for adolescent (10–18 year age combined) in the 3^rd^ BMI quintile. Using this method, the cut-off value of low MFR was 0.374 for Korean boys, and the prevalence of sarcopenia was 0.1%. When we reset the cut-off value of low MFR as mean value– 1 SD of MFR for the 3^rd^ BMI quintile, the MFR cut-off value for sarcopenia was 1.155 for boys and 0.723 for girls, and the prevalence of sarcopenia were 32.1% for boys and 24.3% for girls. In view of the fact that the prevalence of MS is >30% and rapidly increasing in Korea [17], the mean-1SD cutoff appears to be more appropriate for Korean children and adolescents.

Skeletal muscle is the most abundant insulin-sensitive tissue and accounts for 85% of all insulin-mediated glucose utilization [18]. SMM and fat mass have opposing effects on insulin sensitivity and energy disposal, and thus, the presence of low SMM combined with a high fat mass, which would result in low MFR, is likely to compound the risks of MS development. The incidence of MS is increasing worldwide, and MS is known to increase the risk of cardiovascular disease and of type 2 diabetes mellitus [5]. Therefore, the MFR cut-off values used to determine the presence of sarcopenia are clinically important for risk stratification and interventions designed to modify lifestyle factors. BMI is usually used to determine obesity in children and adolescents, but it does not take into account body fat or muscle compositions [19]. Accordingly, we analyzed ORs for MS components for different levels of MFR, and found these risk factors of MS were higher in those with sarcopenia compared with subjects of normal MFR. Furthermore, ORs between MS components and sarcopenia were greater for girls than boys and the ORs were dependent on BMI level. Furthermore, ORs for MS components and body composition were increased progressively from those with high BMI and normal MFR group, and to those with high BMI and low MFR, as those with normal BMI and MFR as reference group. However, the relationship between low MFR and MS risks was insignificant in the normal BMI group. These findings indicate a significant relationship between sarcopenia and risk of MS has a gender and BMI-specific relationship.

This study has several limitations that warrant consideration. First, although the data used was obtained from a representative sample of Korean children and adolescents, the size of the sample could not be sufficient to provide SMM reference values for different age groups. Thus, further large-scale studies are needed to establish appropriate reference values for age- and sex-specific SMM values. Second, since there is no standard cut-off for sarcopenia, we used the mean value—1 SD of MFR for the 3^rd^ BMI quintile to determine sarcopenia in the present study. However, it is evident this cut-off value is somewhat arbitrary and that values are likely to differ in different populations. Third, we analyzed ORs of MS risk components by MFR and BMI levels to test the clinical significances of MFR cut-off values. However, the present study had a cross-sectional design, and BMI and MFR are not used to diagnose MS, but rather to predict metabolic and cardiovascular risk. Accordingly, the clinical significances of our findings should be interpreted cautiously and further prospective studies are needed to verify the predictive nature of the relationship between body composition analysis and MS risks. Finally, this study did not take into account the contribution puberty made to fat mass or fat-free mass, because staging information with respect to puberty was lacking.

Nevertheless, despite these limitations, this is the first study to provide valid measurements of absolute and relative SMMs as determined by DXA in Korean children and adolescents. Th**e** percentile curves obtained show gender- and age- specific changes in SMM. Furthermore, our findings suggest that detailed body composition analyses, including skeletal muscle and body fat, could provide improved measures of metabolic risk.
